# mRNA expression profiles show differential regulatory effects of microRNAs between estrogen receptor-positive and estrogen receptor-negative breast cancer

**DOI:** 10.1186/gb-2009-10-9-r90

**Published:** 2009-09-01

**Authors:** Chao Cheng, Xuping Fu, Pedro Alves, Mark Gerstein

**Affiliations:** 1 Program in Computational Biology and Bioinformatics, Yale University, George Street, New Haven, CT 06511, USA; 2 State Key Laboratory of Genetic Engineering, Institute of Genetics, School of Life Science, Fudan University, Handan Road, Yangpu District, Shanghai, 200433, PR China; 3 Department of Molecular Biophysics and Biochemistry, Yale University, Whitney Avenue, New Haven, CT 06520, USA; 4 Department of Computer Science, Yale University, Prospect Street, New Haven, CT 06511, USA

## Abstract

**Background:**

Recent studies have shown that the regulatory effect of microRNAs can be investigated by examining expression changes of their target genes. Given this, it is useful to define an overall metric of regulatory effect for a specific microRNA and see how this changes across different conditions.

**Results:**

Here, we define a regulatory effect score (RE-score) to measure the inhibitory effect of a microRNA in a sample, essentially the average difference in expression of its targets versus non-targets. Then we compare the RE-scores of various microRNAs between two breast cancer subtypes: estrogen receptor positive (ER^+^) and negative (ER^-^). We applied this approach to five microarray breast cancer datasets and found that the expression of target genes of most microRNAs was more repressed in ER^- ^than ER^+^; that is, microRNAs appear to have higher RE-scores in ER^- ^breast cancer. These results are robust to the microRNA target prediction method. To interpret these findings, we analyzed the level of microRNA expression in previous studies and found that higher microRNA expression was not always accompanied by higher inhibitory effects. However, several key microRNA processing genes, especially *Ago2 *and *Dicer*, were differentially expressed between ER^- ^and ER^+ ^breast cancer, which may explain the different regulatory effects of microRNAs in these two breast cancer subtypes.

**Conclusions:**

The RE-score is a promising indicator to measure microRNAs' inhibitory effects. Most microRNAs exhibit higher RE-scores in ER^- ^than in ER^+ ^samples, suggesting that they have stronger inhibitory effects in ER^- ^breast cancers.

## Abstract 2

Most microRNAs have a stronger inhibitory effect in estrogen receptor-negative than in estrogen receptor-positive breast cancers

## Background

MicroRNAs (miRNAs) are a class of small noncoding (19- to 24-nucleotide) RNAs that regulate the expression of target mRNAs at the post-transcriptional level [1,2]. In higher eukaryotic organisms, it is estimated that miRNAs account for about 1% of genes and regulate the expression of more than 30% of mRNAs [3].

It has been shown that miRNAs play critical roles in a variety of biological processes such as cell proliferation [4], apoptosis [5], development [6], and differentiation [7]. In humans, strong links between cancer and miRNA deregulation have been suggested by recent studies [8,9]. A lot of known miRNAs are found to be located in the fragile sites (regions with high frequencies of copy number alterations in cancers) of human chromosomes, indicating that many miRNAs may be linked to carcinogenesis [10]. Furthermore, it has been shown that aberrant expression of miRNAs contributes to carcinogenesis by promoting the expression of proto-oncogenes or by inhibiting the expression of tumor suppressor genes. For instance, the down-regulation of *let-7*, which represses expression of the proto-oncogene *RAS*, has been found in a large proportion of lung cancer specimens [11]. Other examples are *miR-15 *and *miR-16*, which repress the anti-apoptotic factor gene *BCL2 *in chronic lymphocytic leukemia [12]. In addition, some recent studies suggest that expression profiles of miRNAs are informative for the classification of human cancers. Based on miRNA-expression profiles, Lu *et al*. [13] reported the classification of 334 leukemia and solid cancers that agrees well with the developmental lineage and differentiation state of the tumors. Rosenfield *et al. *[14] demonstrated that by using miRNA as biomarkers, tumors can be classified into subclasses according to their primary origins. Nowadays, miRNAs are thought of as promising biomarkers for cancer diagnosis and prognosis.

It has been proposed that animal miRNAs regulate gene expression mainly by inhibiting translation of their target mRNAs [15,16]. More recent studies, however, have demonstrated that expression regulation at the mRNA level (via mRNA degradation or deadenylation) also serves as a critical mechanism for miRNA function in animals [17-23]. Over-expression of miRNA in cell lines cause moderate down-regulation of a large number of transcripts, many of which contain the complementary sequences of the over-expressed miRNA in their 3' untranslated regions (UTRs) [23]. Conversely, gene expression analysis from miRNA knockdown animals reveals that miRNA recognition motifs are strongly enriched in the 3' UTRs of up-regulated genes, but depleted in the 3' UTRs of down-regulated genes[20]. Motivated by these findings, several studies have demonstrated the effectiveness of investigating miRNA regulation by examining their target mRNA expression levels [24-27]. For example, Yu *et al. *[27] show that miRNA targets have lower expression levels in mature mouse and *Drosophila *tissues than in embryos via global analysis of miRNA target gene expression.

In this study, we investigate differential miRNA regulation between estrogen receptor (ER) positive (ER^+^) and negative (ER^-^) breast cancers by examining changes in the expression of the miRNAs' target genes. Breast cancer is a common disease, ranking first in terms of annual mortality in women worldwide [28]. According to the ER status and responsiveness to estrogen, breast cancer can be divided into two subtypes: ER^+ ^and ER^-^. The links between miRNA expression and breast cancer have been shown using miRNA microarray techniques [13,29]. Specifically, the differential expression of miRNAs between ER^+ ^and ER^- ^breast cancers has been investigated in [30-32]. In comparison with the large number of mRNA expression datasets [33-41], miRNA expression datasets for ER^+ ^and ER^- ^breast cancer are still limited. Moreover, results and conclusions from these studies are generally not consistent and sometimes even conflicting [30-32]. In this study, we take advantage of those mRNA expression datasets to investigate differential miRNA regulation between ER^+ ^and ER^- ^breast cancers.

For each miRNA, we calculate a regulatory effect (RE)-score, which measures the expression difference between the targets and non-targets of the miRNA in an expression profile. Then, we compare the RE-scores of miRNAs in ER^+ ^tumor samples with their RE-scores in ER^- ^samples to identify microRNAs with changing RE-scores (which we term RE-changing microRNAs). We applied our method to five independent microarray datasets that include gene expression profiles for both ER^+ ^and ER^- ^samples. In all of them, our results indicate that the majority of RE-changing miRNAs showed higher RE-scores in ER^- ^than in ER^+ ^samples, suggesting stronger inhibitory effects of miRNAs on their targets in ER^- ^breast cancer. To check the robustness, we performed the same analyses using different miRNA target prediction methods, RE-score calculation methods, and RE-changing miRNA identification thresholds and obtained consistent results. Moreover, we examined the expression levels of genes in the miRNA biogenesis pathway and found that *Ago1 *and *Ago2 *(which encode argonautes, the key proteins forming the RNA-induced silencing complex (RISC)) had significantly higher expression levels in ER^- ^than in ER^+ ^breast cancer. This may suggest higher RISC activities and, therefore, that miRNAs down-regulate target gene expression in ER^- ^breast cancer with higher efficiency.

## Results and discussion

### Identification of RE-changing miRNAs between ER^+ ^and ER^- ^breast cancers

**Figure 1:**
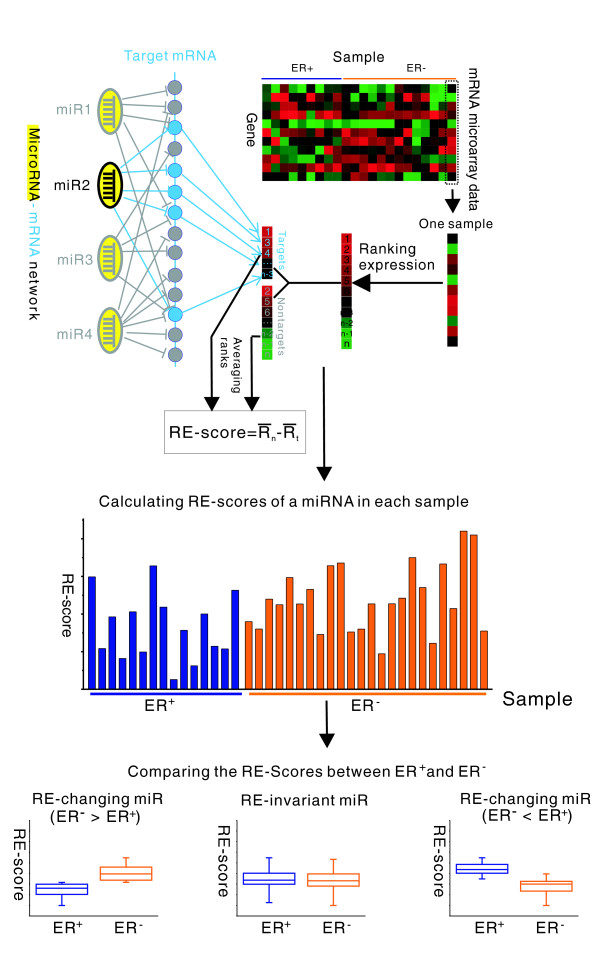
Schematic diagram showing the method for identifying RE-changing miRNAs between ER^- ^and ER^+ ^breast cancer samples. For each miRNA in each sample, a RE-score is calculated by comparing average ranks of its target and non-target genes. RE-changing miR (ER^- ^> ER^+^) and RE-changing miR (ER^- ^< ER^+^) represent miRNAs that have significantly higher and lower RE-scores in ER^- ^compared to ER^+ ^samples, respectively. RE-invariant miR represents miRNAs that show no significant difference in RE-scores between these samples. Note that many miRNAs share the same target mRNA, while many mRNAs can also be targeted by the same miRNA, which constitutes a complex miRNA-mRNA network.

To measure the inhibitory effect of a miRNA, we calculate the RE-score, denoted as the difference of average ranks between the miRNA's non-target and target genes. It should be noted that the RE-scores for different miRNAs may not be directly comparable because the miRNAs regulate different sets of target genes. However, we can compare the RE-scores for the same miRNA in different conditions (that is, using different expression profiles). A higher RE-score indicates lower expression levels of target genes and, thereby, a stronger inhibitory effect of the corresponding miRNA. Given a breast cancer microarray dataset, we calculate the RE-scores for each miRNA in all samples. Then, we compare the RE-scores in ER^+ ^and ER^- ^samples to identify miRNAs that show different regulatory effects between these two breast cancer subtypes. We refer to these miRNAs as RE-changing miRNAs. Using ER^+ ^as the reference, some RE-changing miRNAs show stronger inhibitory effects, while others show weaker inhibitory effects in ER^- ^breast cancer. The false discovery rate (FDR) was estimated using a similar method to the significance analysis of microarrays (SAM) method [42]. A flow diagram of our analysis is shown in Figure 1.

### Most miRNAs show stronger inhibitory effects in ER^- ^than in ER^+ ^breast cancer

**Figure 2:**
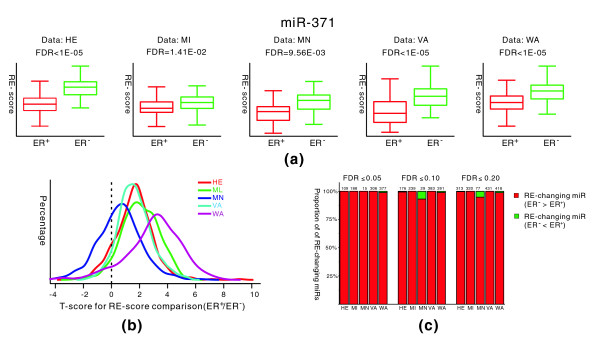
Comparison of RE-scores between ER^+ ^and ER^- ^samples from five breast cancer expression datasets. **(a) **Box plots of RE-scores for *miR-371*. *miR-371 *shows significantly higher RE-scores in ER^- ^than in ER^+ ^samples for all five datasets. The statistical significance level of difference (FDR) for each dataset is also shown. **(b) **Distributions of the t-scores for RE-score comparison between ER^- ^and ER^+ ^samples. The t-scores for 470 miRNAs were calculated by comparing their RE-scores for ER^- ^samples with those for ER^+ ^samples. The t-score distributions for the five datasets are shown in different colors. Most t-scores are positive, indicating that most miRNAs exhibit higher RE-scores in ER^- ^than in ER^+ ^samples. **(c) **Proportion of RE-changing miRNAs with higher inhibitory effect in ER^- ^samples (red) and RE-changing miRNAs with lower inhibitory effect in ER^- ^samples (green) at three different significance levels (FDR ≤ 0.05, FDR ≤ 0.10, and FDR ≥ 0.20). The number on the top of a bar represents how many RE-changing miRNAs were identified from the corresponding mRNA microarray dataset. HE, MI, MN, VA and WA represent the microarray data published by Hess *et al. *[44], Miller *et al. *[38], Minn *et al. *[39], van't Veer et *al. *[34], and Wang *et al. *[40], respectively.

We applied our analysis to 5 carefully selected large scale microarray datasets, each containing at least 30 expression profiles for both ER^+ ^and ER^- ^breast cancer samples. Among these datasets, four were measured by one-channel Affymetrix GeneChips and one was measured by two-channel cDNA arrays (see Materials and methods for details about these datasets). For each dataset, we calculated the RE-scores of each miRNA in all samples. To do this, we needed to determine the target and non-target gene sets for miRNAs. Several computational methods have been developed to identify microRNA targets and predictions using these can be considerably different (Additional data file 1, the distribution of miRNA target gene numbers for different prediction tools). In our analysis, the target genes for miRNAs were predicted using the PITA algorithm, which has been shown to have high prediction accuracy [43]. Subsequently, we computed t-scores (ER^- ^versus ER^+^) to measure the difference between RE-scores for ER^- ^and ER^+ ^samples. A positive t-score for a miRNA suggests that this miRNA has higher overall RE-scores and, thereby, stronger inhibitory effects on its targets in ER^- ^samples. Conversely, a negative ER^-^/ER^+ ^t-score indicates a stronger inhibitory effect of a miRNA in ER^+ ^samples. For example, to estimate the RE-score of *miR-371 *in a sample from the HE (Hess *et al. *[44]) dataset, we first grouped the total 14,327 genes in the HE dataset into two sets, one with 2,054 target genes and the other with 12,273 non-target genes. Second, we sorted the expression levels of the 14,327 genes and computed the average ranks of the 2,054 targets and 12,273 non-targets, respectively. The RE-score for *miR-371 *in each sample was calculated as the average rank of the non-targets minus the average rank of the targets. We performed the RE-score calculation for 82 ER^+ ^samples and 51 ER^- ^samples and found that the RE-scores for the ER^- ^samples are significantly higher than those for ER^+ ^samples (*t*-test, *P *= 3.74E-15). We also compared the RE-scores for ER^+ ^samples with those for ER^- ^samples in the other four datasets. As shown in Figure 2a, in all of the five datasets, the RE-scores for *miR-371 *are significantly higher in ER^- ^samples. Namely, *miR-371 *represses the expression of its target mRNAs more efficiently in ER^- ^breast cancers. In the next section we discuss the results based on other miRNA target prediction methods.

We calculated the ER^-^/ER^+ ^t-scores (measuring the difference between RE-scores for ER^- ^versus ER^+ ^samples) for 470 human miRNAs in all of the 5 datasets. Interestingly, we found that most miRNAs exhibit higher RE-scores in ER^- ^than in ER^+ ^samples, as suggested by the distributions of their t-scores in Figure 2b. We calculated the significance of the t-scores based on the permutation test using a similar method to SAM [42] (see Materials and methods for detail). At the 0.05 significance level (FDR ≤ 0.05), we identified 109, 188, 15 and 306 RE-changing miRNAs from a total of 475 miRNAs in the HE (Hess *et al. *[44]), MI (Miller *et al. *[38]), MN (Minn *et al. *[39]) and VA (van't Veer *et al. *[34]) datasets, respectively, and all of them show higher inhibitory effects in ER^- ^breast cancer. In the WA (Wang et al. [40]) dataset, we identified 377 RE-changing miRNAs, of which 373 have higher inhibitory effects and only 4 lower inhibitory effects in ER^- ^breast cancer. This suggests that most miRNAs exhibit stronger inhibitory effects on the expression of their targets in ER^- ^compared to ER^+ ^breast cancer. This conclusion could still be made when we relaxed the FDR threshold to 10% and 20%, as illustrated in Figure 2c. The t-score, *P*-value and FDR of each miRNA for all datasets are provided in Additional data file 2.

### Use of other miRNA target prediction algorithms

Next, we investigated whether similar results can be obtained using other miRNA target prediction methods. It has been shown previously that distinct miRNA prediction methods may result in considerably different target gene sets (Additional data file 1, the distribution of miRNA target numbers for different prediction tools). To rule out the possible bias introduced by PITA, we repeated our analysis using three other miRNA target prediction methods: TargetScan [3], PicTar [45] and miRanda [46]. We chose these three out of a handful of miRNA target prediction methods not only because they have been prevalently used but also because they are, in some sense, complementary to the PITA method. Almost all miRNA target prediction methods first scan the 3' UTR of transcripts for potential miRNA binding sites that are complementary to the seed region of miRNAs. TargetScan and PicTar meet stringent seed pairing criteria, whereas the criteria are moderately stringent in PITA and miRanda. To further increase the prediction accuracy, PITA takes into account the local accessibility of the potential binding sites, whereas miRanda and PicTar apply a different strategy: they filter out those miRNA binding sites in non-conserved regions. TargetScan, the most widely used prediction method, considers both site conservation and context accessibility.

**Figure 3:**
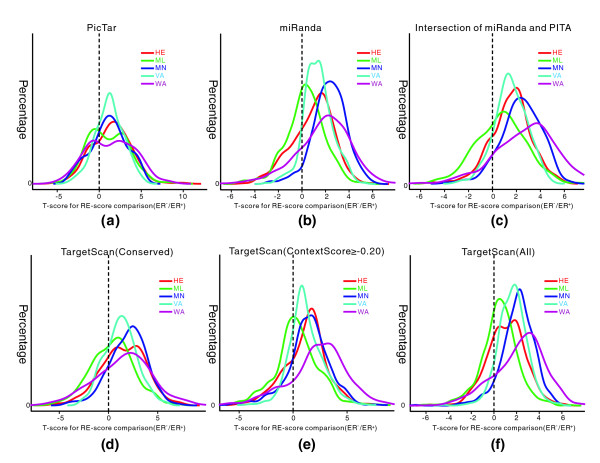
Distributions of the t-scores for comparison of RE-scores based on distinct miRNA target prediction algorithms. **(a) **PicTar algorithm. **(b) **miRanda algorithm. **(c) **Intersection of miRanda and PITA. **(d-f) **TargetScan algorithm where the site is conserved (d), the site context score is above -0.20 (e), and all potential targets are included (f). The t-score distributions for the five datasets are shown in different colors. The t-scores are more likely to be positive values in all five datasets, suggesting that miRNAs have stronger inhibitory effects on their targets in ER^- ^breast cancer.

The results based on PicTar and miRanda are illustrated in Figure 3a, b. As shown, the t-scores for RE-score comparisons for ER^- ^versus ER^+ ^samples are more likely to be positive values for all five datasets, suggesting that miRNAs have stronger inhibitory effects on their targets in ER^- ^breast cancer. Since both PITA and miRanda can require moderately stringent miRNA seed:target complementarity, in order to obtain more reliable target and non-target gene sets for miRNAs, we also tried another strategy: combining the prediction results of PITA and miRanda methods. For each miRNA, we define its target genes as those predicted by both methods and its non-target genes as those predicted by neither. This will presumably decrease both false positive and false negative prediction rates. Based on this target and non-target gene set definition, we again obtain similar results, as shown in Figure 3c.

TargetScan is currently the most widely used microRNA target prediction tool, which relies on strict miRNA seed region complementarity [3,47]. In addition, the conservation of binding site, the context of the miRNA-binding site, the proximal AU composition, and proximity to sites for co-clustered miRNAs can enhance the targeting efficacy of a binding site [48]. Choosing different parameters for target prediction results in quite different performance [49]. Among the parameters, site conservation and site accessibility (measured as context score) are the two most important [50,51]. To evaluate the performance of different TargetScan cutoffs in the RE-score comparison, we chose three target sets - one in which the members have a conserved binding site, one in which the members have a context score greater than -0.20, and one that includes all potential targets - which we refer to as ConservedTS, ContextTS, and AllTS, respectively. These three TargetScan predictions are quite different. On average, 210, 765, and 2,026 targets per miRNA are predicted in ConservedTS, ContextTS and AllTS, respectively. After integrating mRNA expression data with all three target sets to compare the RE-scores in ER^- ^and ER^+ ^samples, we found again that the t-scores for ER^- ^versus ER^+ ^samples are more likely to be positive for all five datasets, as illustrated in Figure 3d-f. This demonstrates that the observation of higher RE-scores in ER^- ^breast cancer, for most miRNAs, is not likely caused by a bias from the miRNA prediction method. Complete results based on three TargetScan predictions, miRanda, PicTar, and the intersection of PITA and miRanda can be found in Additional data file 2.

### Use of alternative methods to compare miRNA inhibitory effects

**Figure 4:**
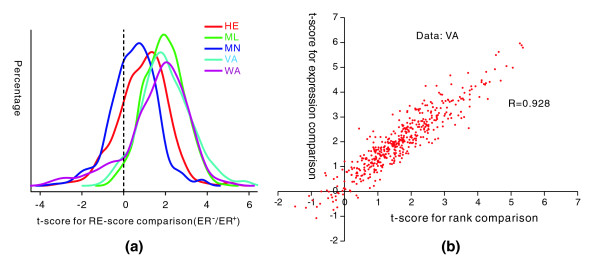
Results obtained from an alternative RE-score calculation method based on expression comparison. **(a) **Distributions of the t-scores calculated by comparing the RE-scores from the expression comparison method. The employed target prediction algorithm was PITA. The t-score distributions for the five datasets are shown in different colors. **(b) **Correlation between the t-scores obtained from the two different RE-score calculation methods. The microarray dataset used was VA, published by van't Veer et *al. *[34]. The correlation coefficient (R) is 0.928, indicating that the t-scores obtained using expression comparison and rank comparison are highly correlated.

To further substantiate our findings, we also used two alternative methods to investigate the inhibitory effects of miRNAs in ER^+ ^and ER^- ^breast cancers. The first method is similar to the one described above, but we use a different way to calculate the RE-scores for miRNAs in an expression profile. Instead of computing the average rank difference between the target and non-target gene sets for a miRNA, we calculate the RE-score as follows: first, calculate the relative expression levels of each gene across all of the samples by subtracting the mean and then dividing by the standard deviation; second, calculate the RE-score of a miRNA by comparing the relative expression levels of its target and non-target genes. For clarity, we will refer to these two RE-score calculation methods as rank comparison and expression comparison. Similar to what we found using the rank comparison method, RE-scores obtained using expression comparison tend to be higher in ER^- ^samples as indicated by the t-score (ER^- ^versus ER^+^) distribution (Figure 4a). These results are not dependent on the miRNA target prediction method because similar results are obtained using PITA and miRanda (complete results are given in Additional data file 3). As a matter of fact, the t-scores obtained by using expression comparison and rank comparison are highly correlated. For example, for the VA dataset, these two methods yield two sets of t-scores with a correlation coefficient of 0.928 (Figure 4b). As shown, 432 out of 466 miRNAs have positive t-scores from both methods, confirming stronger inhibitory effects of miRNAs in ER^- ^breast cancer.

The other method, referred to as ARR (adapted ranked ratio), is similar to the ranked ratio (RR) method proposed by Yu *et al. *[27]. First, the expression levels of each gene in ER^+ ^and ER^- ^samples were compared and a t-score (ER^+^/ER^-^) was calculated to measure the expression differentiation of the gene in the two breast cancer subtypes. The t-scores for all genes were then ranked and genes were divided into two groups, those with high t-scores and those with low t-scores. For each miRNA, the ARR value was calculated by dividing the number of target genes in the 'low' ranked group by the 'high' ranked group. The ARR value is an indicator of the distribution of a miRNA's targets within all genes. A low ARR value (ARR < 1) indicates that a miRNA has more targets in genes with higher t-scores, that is, genes that are lowly expressed in ER^- ^samples; the target genes of this miRNA tend to have lower expression levels in ER^- ^breast cancer. We calculated the ARR values for all miRNAs in each of these five datasets. The numbers of miRNAs with ARR < 1 and ARR > 1 are listed in Table 1. As shown, more miRNAs have ARR < 1 in all datasets, indicating their stronger inhibitory effect in ER^- ^breast cancer.

**Table 1:** Number of miRNAs with ARR < 1 and ARR > 1 in each dataset

	**PITA**			**miRanda**		
		
**Dataset**	**ARR < 1**	**ARR > 1**	**Percentage (ARR < 1)**	**ARR < 1**	**ARR > 1**	**Percentage (ARR < 1)**
HE	279	187	60%	224	188	54%
MI	446	24	95%	380	34	92%
MN	388	79	83%	293	122	70%
VA	407	60	87%	345	71	82%
WA	332	137	71%	299	113	72%

Although the ARR method is similar to the RR method described by Yu *et al. *[27], they differ in some ways. The RR value for a miRNA in a tissue is calculated by dividing the number of targeted genes with 'low' expression by the number of target genes with 'high' expression after the expression levels of each gene across a series of tissues are ranked and split into 'high' and 'low' groups, with half the genes in each. In our ARR method, we first performed a t-test to compare the expression levels of each gene in ER^+ ^and ER^- ^samples. The t-scores were ranked and genes were divided into two groups corresponding to high ranked and low ranked genes, each containing half the genes. The ARR value of each miRNA was then calculated by dividing the number of targets with high rank by the number of targets with low rank. Compared with the RR method described by Yu *et al. *[27], our method is different in three aspects. First, for each gene, the expression levels were compared between ER^+ ^and ER^- ^samples. To reveal the expression difference between two groups, the t-score is more effective than the ranks across all samples. Second, the ARR value from our method is actually an indicator of difference between the expression distribution of a microRNA's target genes and that of all genes. Therefore, it directly reflects the regulatory effect of a microRNA on its target genes. Third, for a microRNA, only one ARR value is obtained based on the whole dataset with our method, and the ARR value facilitates a global inspection of the inhibitory activity differences of a microRNA between two sample groups.

Although the calculations of RE-score and ARR value are completely different, the results from each are highly consistent. We compared the RE-scores determined by expression comparison methods with the ARR results. First, we computed the Spearman correlation of the RE-scores and the ARR values for each microarray dataset. As illustrated in Table 2, the inhibitory activities calculated by these two different methods are highly correlated, with the correlation coefficients ranging from 0.578 to 0.861, which provides further confirmation that more microRNAs show higher inhibitory effects in ER^- ^breast cancers. Second, we overlapped the microRNAs with higher or lower inhibitory activity in ER^- ^cancers predicted by the RE-score and ARR values (Table 3). If a microRNA has a t-score (ER^-^/ER^+^) > 0 in the RE-score comparison and ARR < 1 in the ARR calculation, it is predicted to have higher inhibitory activity in ER^- ^cancer by both methods, whereas a microRNA with a t-score < 0 and RR > 1 shows consistently higher activity in ER^+ ^cancer. More than 80% of the miRNAs overlap, indicating that these two methods are in strong agreement. Furthermore, the number of miRNAs with consistently higher activity in ER^- ^samples is much higher than the number with consistently lower activity in ER^+ ^samples, again indicating that most miRNAs exhibit higher regulatory effects in ER^- ^than in ER^+ ^samples. Some significant miRNAs are identified by both methods. For example, it has been reported that *miR-206*, which regulates the estrogen receptor, has higher activity in ER^- ^than ER^+ ^cancers [52]. In our calculations, for all five microarray datasets, the ARR values of this microRNA are all <1, and the t-scores for RE-score comparison between ER^- ^and ER^+ ^cancers are all >0 (Table 3). These results are consistent with the activity difference between ER^+ ^and ER^- ^cancer reported by Adams *et al. *[52].

**Table 2:** Correlation between the results obtained using the ARR and RE-score calculation methods

	**PITA**			**PicTar**		
		
**Dataset**	**Percentage (ER^- ^> ER^+^)**	**Percentage (ER^- ^< ER^+^)**	**Spearman correlation**	**Percentage (ER^- ^> ER^+^)**	**Percentage (ER^- ^< ER^+^)**	**Spearman correlation**
HE	58%	23%	0.861	77%	12%	0.752
MI	100%	0%	0.646	60%	16%	0.778
MN	73%	10%	0.668	62%	17%	0.763
VA	86%	2%	0.659	65%	5%	0.578
WA	68%	18%	0.855	59%	22%	0.837

Percentage (ER^- ^> ER^+^): the fraction of microRNAs with ARR < 1 and t-score < 0, indicating that the microRNAs show higher regulatory activity in ER^- ^than in ER^+ ^samples, as consistently supported by both the ARR method and RE-score expression comparison method. Percentage (ER^- ^< ER^+^): the fraction of microRNAs with RR > 1 and t-score > 0. These microRNAs show higher regulatory activity in ER^+ ^samples, as supported by both the ARR method and the RE-score expression comparison method. Spearman correlation: the correlation between the ARR value and t-score (ER^-^/ER^+^).

**Table 3:** Regulatory activity of *miR-206 *predicted by the RE-score and ARR methods

	**PITA**		**PicTar**	
		
**Dataset**	**ARR**	**t-score (ER^-^/ER^+^)**	**RR**	**t-score (ER^-^/ER^+^)**
HE	0.986	0.91	0.852	1.98
MI	0.973	1.91	0.977	1
MN	0.97	1.02	0.783	2.56
VA	0.979	2.47	0.823	3.82
WA	0.977	1.57	0.84	3.09

t-score (ER^-^/ER^+^): the t-score is calculated by performing a *t*-test to measure differentiation of the RE-scores for a miRNA in the two breast cancer subtypes. Note that here the RE-scores were calculated using the expression comparison method.

### Differential regulatory effects of miRNAs can not be explained by miRNA expression differences between ER^+ ^and ER^- ^cancer

To understand why miRNAs tend to have stronger inhibitory effects on their targets in ER^- ^samples, we asked whether they are more highly expressed in ER^- ^breast cancers. Using miRNA microarray technology, expression levels of miRNAs have been previously measured and compared in ER^- ^and ER^+ ^samples in three different studies [30-32]. Iorio *et al. *[31] identified 11 miRNAs that were differentially expressed between ER^+ ^and ER^- ^samples, of which 8 were down-regulated in the ER^- ^samples. In contrast, many more miRNAs were reported to be differentially expressed by Blenkiron *et al. *[30] and Mattie *et al. *[32]. Specifically, Blenkiron *et al. *identified 35 differentially expressed miRNAs, of which 11 were up-regulated and 24 were down-regulated in the ER^- ^samples. Mattie *et al.*, however, reported that the majority of differentially expressed miRNAs were down-regulated in ER^- ^samples (40 out of 43). These three miRNA expression studies do not support the idea that miRNAs tend to be more highly expressed in ER^- ^than ER^+ ^breast cancer. It should be noted that the three studies obtained substantially different results due to the technological issues of miRNA microarray experiments.

In addition, to measure the correlation between miRNAs' inhibitory effects and their expression levels, we calculated the Spearman correlations of the t-scores for the miRNA expression comparisons and those for the miRNA RE-score comparisons. As illustrated in Table 4, there is only a very weak positive correlation between them; particularly, the miRNA expression data published by Mattie *et al. *[32] shows almost no correlation with the miRNA regulatory effects predicted from all five mRNA expression datasets. This further indicates that the stronger inhibitory effect of miRNAs in ER^- ^cancer cannot be explained by their expression levels.

**Table 4:** Correlation between microRNA RE-scores and their expression levels

	**Expression level**			
	
	**PITA**		**miRanda**	
		
	**BL**	**MA**	**BL**	**MA**
RE-score				
HE	0.218	0.023	0.150	-0.069
MI	0.211	0.056	0.254	-0.072
MN	0.235	0.089	0.201	-0.085
VA	0.102	0.015	0.071	-0.044
WA	0.285	0.121	0.251	-0.055

BL and MA represent the microRNA microarray data published by Blenkiron *et al. *[30] and Mattie *et al. *[32]. HE, MI, MN, VA and WA represent the mRNA microarray data published by Hess *et al. *[44], Miller *et al. *[38], Minn *et al. *[39], van't Veer *et al. *[34], and Wang *et al. *[40], which were used to infer the microRNA RE-scores.

**Figure 5:**
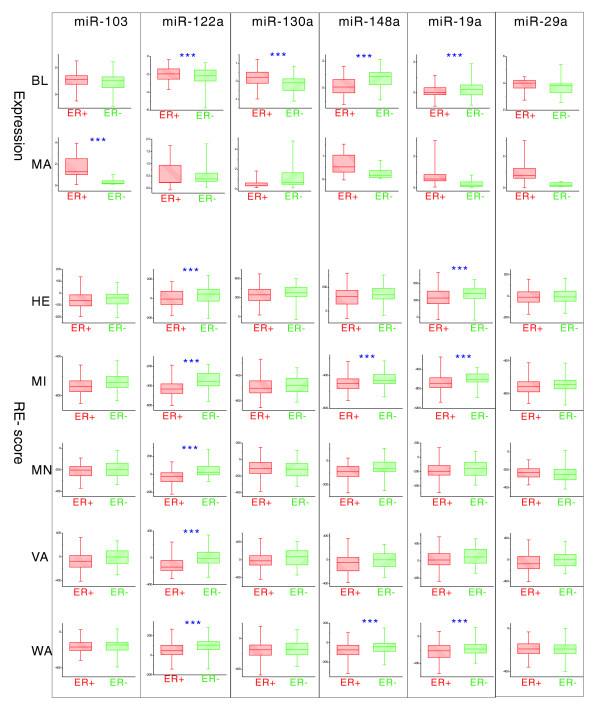
The expressions and RE-scores of microRNAs predicted to target *Dicer*. BL and MA represent the microRNA microarray data published by Blenkiron *et al. *[30] and Mattie *et al. *[32]. HE, MI, MN, VA and WA represent the mRNA microarray data published by Hess *et al. *[44], Miller *et al. *[38], Minn *et al. *[39], van't Veer et *al. *[34], and Wang *et al. *[40], which were used to calculate the microRNA RE-scores. If the difference between ER^+ ^and ER^- ^samples is significant, the plot is flagged with three asterixes. The expression levels of these six microRNAs are mostly lower in ER^- ^samples; however, almost all the RE-scores in ER^- ^samples are higher, suggesting that the differential regulatory effects of miRNAs can not be explained by miRNA expression difference between ER^+ ^and ER^- ^cancers.

Some microRNAs have large inconsistencies between their expression levels and RE-scores. For example, many studies have suggested that the expression levels of *Dicer*, the key gene in the generation of microRNAs, vary in different cancer subtypes [53-55]. In our study, *Dicer *is significantly down-regulated in ER^- ^compared to ER^+ ^cancers (see next section for details). A possible mechanism for this is that it is regulated epigenetically [56]. Six microRNAs, *miR-103*, *miR-122a*, *miR-130a*, *miR-148a*, *miR-19a*, and *miR-29a*, are commonly predicted to target *Dicer *by the prediction methods PITA, miRanda, PicTar and Targetscan. We investigated the expression levels of these microRNAs in two distinct datasets published by Blenkiron *et al. *[30] and *Mattie et al. *[32]. The expression levels of these microRNAs are mostly lower in ER^- ^samples (Figure 5), which is opposite to our inference that they may be up-regulated to transcriptionally repress *Dicer *in ER^- ^cancer. We then compared the RE-scores of these microRNAs in ER^+ ^and ER^- ^cancers. To our surprise, almost all microRNAs show stronger inhibitory effects in ER^- ^cancers (Figure 5), which may explain why *Dicer *is expressed less in ER^- ^cancer. Especially, *miR-122a*, which was reported to target *Dicer *and function in various cellular stresses [57,58], is expressed at significantly lower levels but shows significantly higher inhibitory activity in ER^- ^cancer, strongly indicating that the differential regulatory effects of miRNAs can not be explained by miRNA expression differences between ER^+ ^and ER^- ^cancer.

**Figure 6:**
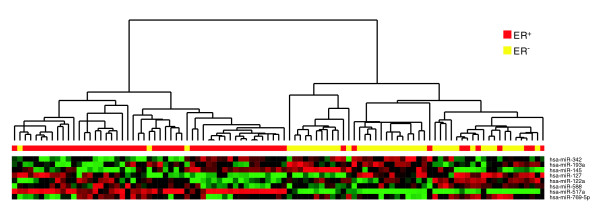
RE-score profiles of microRNAs for the classification of ER^+ ^and ER^- ^breast tumors. The figure demonstrates unsupervised hierarchical clustering of 57 ER^+ ^and 42 ER^- ^samples in the MN dataset [39] using the top 8 RE-changing miRNAs. A dendrogram of the tumors is shown at the top, with ER^+ ^samples in red and ER^- ^samples in yellow. For hierarchical clustering, RE-scores of each miRNA were mean centered and normalized, and tumors were clustered using Pearson correlation (uncentered) and average linkage (CLUSTER and TREEVIEW software) [73].

Several studies have reported that good classification of cancer subtypes can be achieved using the expression levels of miRNAs [13,14]. Because striking differences in the RE-scores for a set of miRNAs between ER^+ ^and ER^- ^samples are observed, the RE-score of an miRNA could be a promising predictor for breast cancer subtype classification. We used the RE-scores of the top eight significantly RE-changing miRNAs in the MN dataset [39] to classify the ER^+ ^and ER^- ^subtypes. As expected, the accuracy was up to 89.29%. The RE-score profiles of these miRNAs are plotted in Figure 6. The classification accuracy was comparable or even better (85.76%) when estimated using the expression levels of the top 35 differentially expressed miRNAs in the dataset published by Blenkiron *et al. *[30], suggesting that the prediction of ER status of breast cancer based on miRNA regulatory effect or miRNA targeted mRNA expression is an alternative to that based on miRNA expression.

### Differential expression of miRNA processing genes between ER^+ ^and ER^- ^breast cancers

In addition to miRNA abundance, post-transcriptional regulation of miRNA expression may also be important for the inhibitory effect of miRNAs on their targets. Deregulation of genes required for miRNA biogenesis may be expected to lead to global changes in miRNA expression as well as the inhibitory effects of miRNAs. Therefore, we examined whether miRNA processing genes are differentially expressed in ER^+ ^and ER^- ^breast cancers.

**Figure 7:**
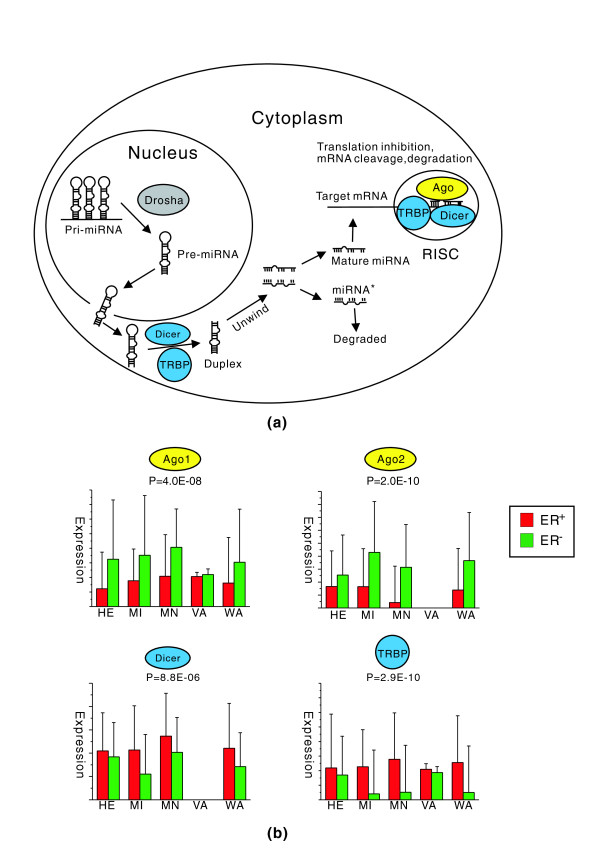
Differential expression of miRNA processing genes between ER^+ ^and ER^- ^breast cancer samples. **(a) **miRNA biogenesis and function pathway. Genes significantly up-regulated and down-regulated in ER^- ^compared to ER^+ ^samples are shown in yellow and cyan, respectively. A gene - for example, *Drosha *- is marked grey to denote that it shows no significant differential expression between ER^- ^and ER^+ ^samples. **(b) **Expression levels of *Ago1*, *Ago2*, *Dicer*, and *TRBP *in ER^+ ^(red) and ER^- ^(green) samples. The mean and the standard deviation of the expression levels for each gene are shown as a bar and vertical line, respectively. Data for a gene are not shown if it is missing from a dataset. The combined *P*-value for each gene is also shown.

We found that among the miRNA processing genes, *Ago1 *and *Ago2 *were significantly up-regulated in ER^- ^compared to ER^+ ^samples in all datasets, with combined *P*-values of 4.0E-8 and 2.0E-10, respectively, whereas *Dicer *and *TRBP *were significantly down-regulated, with combined *P*-values of 8.8E-6 and 2.9E-10, respectively (Figure 7b; Additional data file 4). Differential expression of *Ago1*, *Ago2 *and *Dicer *between ER^+ ^and ER^- ^breast cancer has been previously investigated and consistent results were reported by Blenkiron *et al. *[30]. As shown in Figure 7a, several proteins play a critical role in the miRNA processing pathway. DROSHA, a double-stranded RNA-specific ribonuclease, digests the pri-miRNA in the nuclease to release hairpin, precursor miRNA (pre-miRNA) [7]; then DICER, a member of the RNase III nuclease, cleaves the pre-miRNA into a single-stranded mature miRNA with the assistance of TRBP [59]; finally, the mature miRNA is incorporated into RISC consisting of DICER, TRBP, AGO and several other proteins [60-62]. Among the eight human AGO proteins, AGO1 and AGO2 are known to play the most important roles in transcriptional silencing mediated by miRNAs or small interfering RNAs. Assembly of human RISC minimally requires AGO2, DICER, and TRBP, among which AGO2 is the catalytic engine owing to its endonuclease activity and the DICER-TRBP complex acts simply as a platform [60,63,64]. The relatively lower abundance of AGO1 and AGO2 proteins in ER^+ ^breast cancer may limit the activity of functional RISC, which would in turn lower the inhibitory effect of miRNAs on their targets. Moreover, since the expression levels of *Dicer *and *Ago *genes are anti-correlated in ER^+ ^and ER^- ^cancer, there is no necessary link between the mature miRNA expression levels and RISC activity. This may also explain the global up-regulation of miRNA expression levels in ER^+ ^cancer observed by Blenkiron *et al. *since *Dicer *is significantly up-regulated [30].

It seems that the key genes in the microRNA biogenesis pathway are subjected to delicate regulation and their differential expression is likely to be associated with distinct tumor subtypes. More interestingly, genes in this pathway are not consistently regulated: *Dicer *and *TRBP*, which are involved in miRNA maturation and RISC assembly, are down-regulated whereas the catalytic engine of RISC is up-regulated in ER^- ^relative to ER^+ ^breast cancer. As a result, the capability of miRNAs (or more precisely RISC) to repress their targets may not be reflected by their expression levels. Using microRNA microarray experiments, Blenkiron *et al. *[30] found that the most differentially expressed miRNAs between ER^+ ^and ER^- ^cancers are down-regulated in the latter. They also examined the correlation between miRNA expression and changes in the mRNA levels of their direct targets but failed to detect enrichment for down- or up-regulation of predicted target miRNAs consistent with miRNA expression differentiation in most cases. This can be explained by the hypothesis that many miRNAs act at the level of translation rather than mRNA stability; nevertheless, this can also be explained by discordance in changes of expression between the key miRNA processing genes. Our results demonstrate that miRNAs tend to have stronger inhibitory effect on their mRNA targets in ER^- ^breast cancer, suggesting that the AGO proteins (up-regulated in ER^- ^cancer at the mRNA level), the catalytic engine of RISC, may eventually determine the efficiency of miRNAs to down-regulate their targets. In addition, deregulation of the key genes in the miRNA biogenesis pathway may be related to tumorigenesis of certain cancer types, as has been suggested by the fact that down-regulating DICER expression promoted tumorigenesis *in vitro *and in a mouse lung cancer model [65].

It has been reported that *Ago2 *is expressed more in ER^- ^than in ER^+ ^breast cancer cell lines, and that this is correlated with active ER signaling [66]. AGO2 enhances cell proliferation, reduces cell-cell adhesion, and increases cell migratory ability, which contribute to the tumor phenotype transformation from ER^+ ^to ER^- ^through overexpression of *Ago2*. Either gene amplification or activation of cell signaling cascades elevates *Ago2 *expression in ER^- ^cancer cells. Up to now, no clear evidence or experimental data have shown that *Ago2 *is amplified in ER^- ^cancer. The epidermal growth factor receptor (EGFR) and mitogen activated protein kinase (MAPK) signaling cascades are the major signal transduction pathways in ER^- ^breast cancers [67,68]. One of the frequent and remarkable features in ER^- ^cancer is the up-regulated *EGFR *gene [69]. Adams *et al. *[66] proposed and confirmed that epidermal growth factor stimulated *Ago2 *expression in ER^- ^cancers and that this was primarily regulated by the MAPK pathway. In addition, with the overexpression of *Ago2*, the inhibition activity of *miR-206 *was elevated, whereas without *Ago2 *the activity of *miR-206 *remained unchanged even with the overexpression of *miR-206*, suggesting that formation of Ago2-miRNA complexes is the main factor influencing *miR-206 *inhibitory activity [70]. This is consistent with our finding that the activity of a microRNA cannot be explained merely by its expression level. Based on this suggestion, a hypothesis can be provided that, with elevated *Ago2 *expression, an miRNA's inhibitory activity accordingly increases, which leads to low expression levels of genes involved in ER^+ ^cell types and the predominant expression of genes involved in the oncogenic pathways leading to ER^- ^cancer.

Despite growing evidence that *Dicer *mRNA levels vary between different tumor subtypes and that these variations are correlated with cancer progression [53-55], the regulation of *Dicer *remains unclear. Weisen *et al. *[56] reported that type I interferon represses *Dicer*. As already reported, MAPK signaling pathways comprise a major cascade in ER^- ^cancers [68]. Type I interferon signals can be transduced by the MAPK pathway [71], and the activated MAPK pathway in ER^- ^cancers may enhance the signal of type I interferon, which results in the inhibition of *Dicer *expression. Another possible explanation of the low expression of *Dicer *in ER^- ^cancers may be the regulatory effect of miRNAs. DICER's epigenetic regulation could also occur via specific mechanisms involving the DICER 3' UTR and the binding of microRNAs [56]. In this study, we have shown that the activity of miRNAs is stronger in ER^- ^than ER^+ ^cancer and that *Dicer *is targeted and suppressed to a lower level in ER^- ^compared to ER^+ ^cancers.

## Conclusions

In this study, we created the RE-score to measure the inhibitory effect of a miRNA on its targets. Based on RE-score calculations, we compared the inhibitory effects of miRNAs on their targets between two breast cancer subtypes, ER^+ ^and ER^-^. miRNAs that showed significantly different inhibitory effects were identified for five independent datasets. We found that, for most miRNAs, the target genes were more repressed in ER^- ^than ER^+ ^breast cancer, suggesting that miRNAs have stronger inhibitory abilities in the former. The exact identity of the miRNA targets does not seem important since these findings are robust to several distinct methods of miRNA target prediction and are further consolidated by another two methods for comparing miRNA regulation. To seek the potential mechanisms contributing to the inhibitory effects of miRNAs, we explored miRNA abundance measured by miRNA microarrays and expression levels of genes involved in miRNA biogenesis and function. Our analysis indicates that a high inhibitory ability is not necessarily associated with high miRNA expression levels, because previous miRNA expression data do not suggest prevalent over-expression in ER^- ^breast cancer. However, it is interesting to find that several key miRNA processing genes are significantly differentially expressed between ER^+ ^and ER^- ^breast cancer. *Ago1 *and *Ago2 *are significantly up-regulated in ER^- ^cancer, while *Dicer *and *TRBP *are significantly down-regulated. These results imply that the miRNA processing pathway is subject to subtle regulation and that deregulation of key genes in this is involved in the cancer pathology. This method is easily applied and can be used to investigate the miRNA regulation underlying other microarray datasets.

## Materials and methods

### Breast cancer microarray datasets

All the microarray data used in this study were downloaded from public databases or from the websites provided by the original publications. Over ten breast cancer datasets have been generated in previous studies [33-41,44]. From these datasets, we chose five according to the following criteria: contain at least 30 samples for both ER^+ ^and ER^- ^breast cancer; and expression of ER^+ ^and ER^- ^samples is measured using the same platform. The first criterion is to ensure a high power of statistical analysis, while the second criterion is to avoid bias introduced by platform effect. Among these five datasets, one used cDNA arrays and the other four used oligonucleotide arrays produced by Affymetrix. Numbers of ER^+ ^and ER^- ^samples in each dataset are listed in Table 5. The expression values are represented by normalized log ratios for cDNA microarrays or by log-transformed intensities after Robust Multichip Average normalization for Affymetrix oligonucleotide microarrays [72]. The probe or probeset IDs are mapped to NCBI Refseq IDs. When multiple probe sets are mapped to the same Refseq ID, their values are averaged to represent the expression level of this Refseq gene.

**Table 5:** Breast cancer gene expression datasets used in this study

			**Number of samples**	
			
**Dataset ID**	**Reference**	**Array type**	**ER^+^**	**ER^-^**
HE	Hess *et al. *[44]	One channel oligo	82	51
MI	Miller *et al. *[38]	One channel oligo	213	34
MN	Minn *et al. *[39]	One channel oligo	57	42
VA	van't Veer *et al. *[34]	Two channels cDNA	53	44
WA	Wang *et al. *[40]	One channel oligo	209	77

### miRNA target predictions

A number of miRNA target prediction approaches have been suggested in the past few years [43,45-47]. In this paper, we utilize four sets of miRNA target prediction data derived using PITA [43], miRanda [46], PicTar [45] and TargetScan [3,47], respectively. All miRNA target prediction datasets were downloaded from the most recently updated websites. To facilitate the analysis, the target gene IDs were also converted into NCBI Refseq IDs. For each miRNA, the target genes are defined as those presented in the microarray data and predicted to contain at least one binding site at their 3' UTR; the non-target genes are defined as those presented in the microarray data but not predicted to be regulated by the miRNA.

### Measuring a miRNA's inhibitory effect with the average rank difference between its targets and non-targets

To measure the inhibitory effect for a miRNA, we defined the RE-score, which measures the difference in expression levels between its target and non-target genes. The RE-score can be calculated in two ways: one is based on rank comparison and the other is based on expression comparison.

The RE-score based on rank comparison is calculated as follows. We denote the number of a miRNA's targets and non-targets as *N*_*t *_and *N*_*n*_, respectively. After sorting the expression levels of all genes, the ranks of target genes and a non-target genes are denoted as *R*_*t *_and *R*_*n*_, respectively. The RE-score of a miRNA is defined as the difference of the average rank between its targets and non-targets:



where  and  represent the mean target and non-target ranks, respectively. The RE-score is essentially a transformation of the sum rank statistic (the sum of ranks for target genes) used in the Wilcoxon rank sum test. Since genes with high absolute expression values have high rank values, a positive RE-score indicates that the non-target genes of a miRNA tend to be expressed at higher levels than its target genes, presumably due to the inhibitory effect of the miRNA on its target genes. The higher the RE-score, the stronger the inhibitory effect of a miRNA on its targets.

### Identifying microRNAs with significantly changed RE-scores between ER^+ ^and ER^- ^breast cancer

To investigate the difference of a miRNA regulatory effect between ER^+ ^and ER^- ^breast cancer, a two sample *t*-test was performed to compare RE-scores and determine whether the RE-scores of a miRNA are significantly different between ER^+ ^and ER^- ^cancer.

Since usually hundreds of miRNAs are examined simultaneously, multiple testing corrections needed to be considered. We calculated the FDR based on permutations similar to the method used in SAM [42]. If there were N_1 _ER^+ ^and N_2 _ER^- ^samples, the t-scores obtained from comparing RE-scores for each miRNA were calculated in the original data, denoted as T_RES_(r) for the r^th ^miRNA. We then permutated the samples; at each permutation, N_1 _samples were randomly selected to form one permuted ER^+ ^group, and the rest of the samples were used as the permutated ER^- ^group. The permutated t-score, denoted as T_RES_(r, k), for the r^th ^miRNA in the k^th ^permutation, is recalculated. We then considered the histogram of all T_RES_(r, k) over all r and k, and used this null distribution to compute an FDR value for a given t-score T_RES_(r) = T_RES_*. If T_RES_* ≥ 0, the FDR is the ratio of the percentage of all (r, k) with T_RES_(r, k) ≥ 0, whose T_RES_(r, k) ≥ T_RES_*, divided by the percentage of miRNAs with T_RES_(r) ≥ 0, where T_RES_(r) ≥ T_RES_*, and similarly if T_RES_* < 0.

If the FDR for a miRNA is below a predefined threshold, we call this miRNA as a significantly RE-changing miRNA.

### ER^+ ^and ER^- ^cancer subtype classification

The miRNA RE-score is a promising feature to classify tumor subtypes as well as microRNA expression. In this study, we constructed a multi-miRNA signature and used the algorithm of a linear support vector machine. We ranked the *P*-values that were derived from the comparison of RE-scores or expressions. The top N significant RE-score changing microRNAs or differentially expressed microRNAs were chosen to perform the classification analysis. To estimate the effect of the classifier, we adapted a leave one out cross validation strategy. Generally, the number of ER^+ ^samples is different from the number of ER^- ^samples. If there were N_1 _ER^+ ^and N_2 _ER^- ^samples, assuming that N_1 _> N_2_, in order to balance the sample effect, we randomly selected N_2 _ER^+ ^samples. The total 2* N_2 _(N_2 _ER^+ ^and N_2 _ER^-^) samples were used in the leave one out validation. The classification accuracy was determined by averaging the accuracies of the leave one out validations repeated 100 times.

## Abbreviations

ARR: adapted ranked ratio; ER: estrogen receptor; FDR: false discovery rate; MAPK: mitogen activated protein kinase; miRNA: microRNA; RE: regulatory effect; RISC: RNA-induced silencing complex; RR: ranked ratio; SAM: significance analysis of microarrays; UTR: untranslated region.

## Authors' contributions

CC and MG conceived and designed the study. CC extracted the gene expression data. CC and XF preformed the full analysis. CC, XF, PA and MG wrote the manuscript.

## Additional data files

The following additional data are available with the online version of this paper: a figure showing distributions of miRNA target numbers determined using different prediction tools (Additional data file 1); a table listing RE-score results for five breast cancer expression datasets (Additional data file 2); a table listing RE-score results calculated using the expression comparison method (Additional data file 3); a table listing expression levels of several miRNA processing genes in ER^+ ^and ER^- ^samples (Additional data file 4).

## Supplementary Material

Additional data file 1 Distribution of miRNA target numbers for four prediction tools, PITA, miRanda, PicTar, and TargetScan. In addition, three different parameters in TargetScan were chosen and are denoted as 'Conserved', 'ContextScore ≥ -0.20' and 'All', respectively. On average, 6,949, 2,026, 1,563, 765, 426, and 210 targets per miRNA were predicted by PITA, TargetScan(All), miRanda, TargetScan(ContextScore ≥ -0.20), PicTar, and TargetScan(Conserved), respectively. Click here for file

Additional data file 2 Includes seven sheets containing the complete results based on RE-scores from rank comparison and seven miRNA target predictions from PITA, miRanda, PicTar, the intersection of PITA and miRanda, TargetScan(Conserved), TargetScan(ContextScore ≥ -0.20), and TargetScan(All), respectively. The t-score, *P*-value, and adjusted *P*-value (FDR) of all miRNAs in the five breast cancer datasets are provided. Click here for file

Additional data file 3 Additional data file 3 includes two sheets containing the complete results based on RE-scores calculated from expression comparison. In the two sheets, miRNA target predictions determined by the miRanda and PITA tools are used, respectively. Click here for file

Additional data file 4 Expression levels of several miRNA processing genes in ER^+ ^and ER^- ^samples. Click here for file
