# Supplementary figures and images for: mRNA expression profiles show differential regulatory effects of microRNAs between estrogen receptor-positive and estrogen receptor-negative breast cancer
Source: Genome Biol. 2009 Sep 1;10(9):R90. doi: 10.1186/gb-2009-10-9-r90 (PMC2768979; doi:10.1186/gb-2009-10-9-r90)

The distributions of target number for different predictions

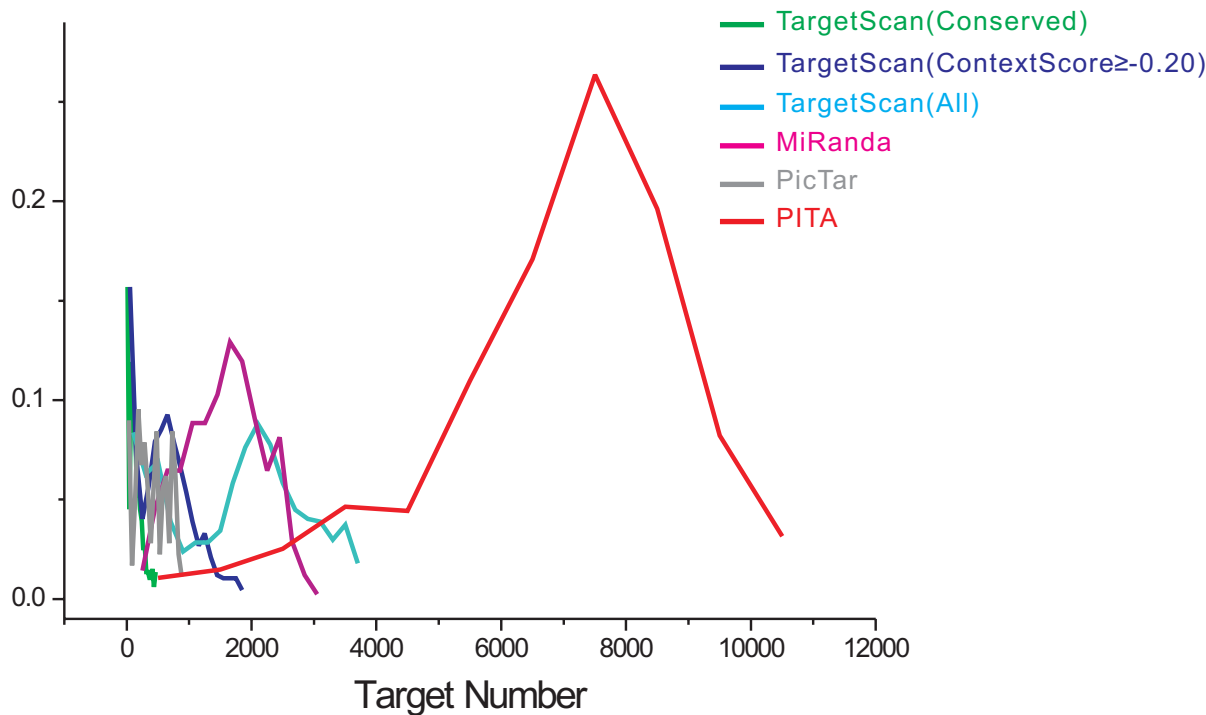

Supplement: Additional data file 1 — Distribution of miRNA target numbers for four prediction tools, PITA, miRanda, PicTar, and TargetScan. In addition, three different parameters in TargetScan were chosen and are denoted as 'Conserved', 'ContextScore ≥ -0.20' and 'All', respectively. On average, 6,949, 2,026, 1,563, 765, 426, and 210 targets per miRNA were predicted by PITA, TargetScan(All), miRanda, TargetScan(ContextScore ≥ -0.20), PicTar, and TargetScan(Conserved), respectively. [file gb-2009-10-9-r90-S1.pdf]
